# Supplementary material for: The interactome of intact mitochondria by cross-linking mass spectrometry provides evidence for coexisting respiratory supercomplexes
Source: Mol Cell Proteomics. 2017 Dec 8;17(2):216–32. doi: 10.1074/mcp.RA117.000470 (PMC5795388; doi:10.1074/mcp.RA117.000470)
Supplement: Supplemental Data [file supp_RA117.000470_133922_0_supp_23978_fzffwf.zip › spectra_annotation/mito_DR_spectra_annotation/81-1-2-1-14-1.pdf]

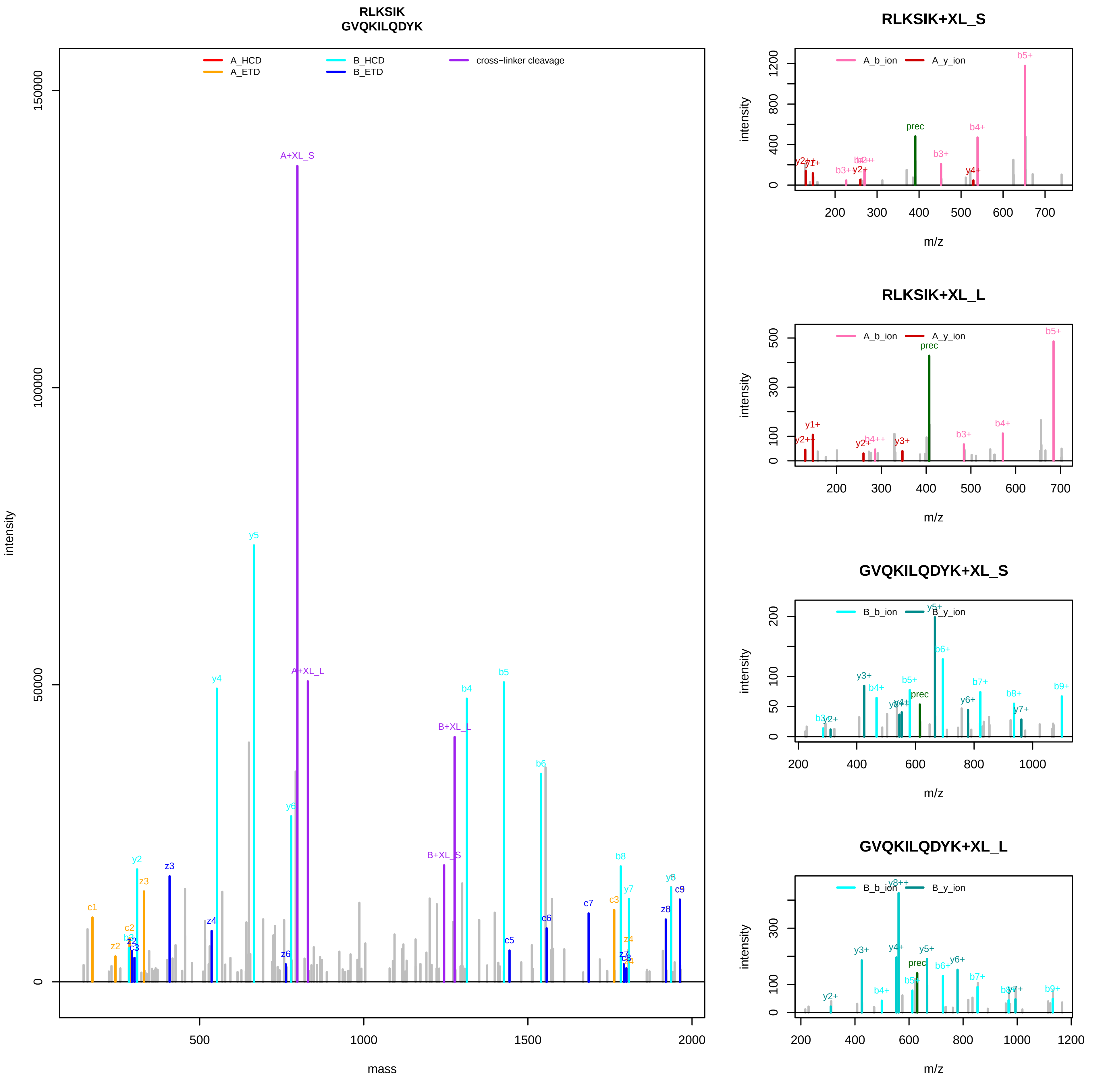

RLKSIK
GVQKILQDYK
A_HCD
A_ETD
B_HCD
B_ETD
cross−linker cleavage
intensity
mass
0
50000
100000
150000
500
1000
1500
2000
A+XL_S
A+XL_L
B+XL_L
B+XL_S
y2
y4
y5
y6
y7
y8
b3
b4
b5
b6
b8
c1
c2
c3
c5
c6
c7
c8
c9
z2
z3
z4
z6
z7
z8
RLKSIK+XL_S
A_b_ion
A_y_ion
prec
b3+
b4+
b5+
y1+
y2+
y4+
m/z
RLKSIK+XL_L
b4++
y3+
GVQKILQDYK+XL_S
B_b_ion
B_y_ion
y3+
y5+
y6+
y7+
b6+
b7+
b8+
b9+
GVQKILQDYK+XL_L
y8++
